# Functional Capacity and Levels of Physical Activity in Aging: A 3-Year Follow-up

**DOI:** 10.3389/fmed.2017.00244

**Published:** 2018-01-09

**Authors:** Maria Teresa Tomás, Alejandro Galán-Mercant, Elvis Alvarez Carnero, Beatriz Fernandes

**Affiliations:** ^1^Escola Superior de Tecnologia da Saúde de Lisboa (ESTeSL), Instituto Politécnico de Lisboa, Lisboa, Portugal; ^2^Health Science Department, Jaén University, Jaén, Spain; ^3^Translational Research Institute for Metabolism and Diabetes, Florida Hospital, Orlando, FL, United States

**Keywords:** predictors of disability, functional capacity, physical activity levels, aging, handgrip, 6 min walk test

## Abstract

Over the last decades, the world elderly population has increased exponentially and this tendency will continue during the coming years; from 2000 to 2050, people over 60 will double and those over 80 will quadruple. Loss of independence occurs as people age due to mobility restrictions, frailty, and decreased functional fitness and cognitive abilities. Evidence has shown that appropriate programs and policies contribute to keep older adults healthy and independent over time. The purpose of this chapter is to report the results of our 3-year follow-up study designed to characterize functional physical fitness in a sample of Portuguese community-dwelling older adults to propose a set of functional parameters that decline the most. We studied a group of 43 elderly people, aged 60 and over. Variables assessed on the participants were anthropometric measurements, functional capacity with the Senior Fitness Test battery (muscle strength, aerobic endurance, flexibility, agility, and dynamic balance), handgrip strength, levels of physical activity, and balance. Three years after the first assessment, a second assessment of the same variables was conducted. We analyzed what were the variables that, for this group, were related with a healthier aging and the relation with different physical activity levels. Our study showed that the distance covered in 6-min walk test and handgrip strength seem to explain a great amount of variability on functional variables that have changed on this period (68% of balance, lower and upper functional strength, respectively) and the active participants showed less decrements with aging in anthropometric and functional variables than those inactive or insufficiently active (*p* < 0.05). Greater importance should be given to prescription of exercise targeting older adults and, specifically, walking and manual activities should be given more attention as components of a community exercise program.

## Introduction

Aging is a gradual, life-long process and highly variable characterized by a progressive and cumulative generalized impairment of physiological functions (1), which may be explained at least by genetic factors, multiple morbidities, and non-genetic factors (specially nutrition, lifestyle, and physical activity) (1).

Nowadays, the increase in human life expectancy and the reduction in death rates has made the number of older adults to grow. Portugal is not an exception and, the rate of older people has risen until 148.7% in 2016 (2), which makes authorities and health care professionals be concerned with the retirement impact on levels of functional limitations (3).

According to Chen and colleagues, it is important to study disability as a process over time since it is very informative on the disability process of an aging population (4). So, a comprehensive follow-up of functional impairment rates and its relationship with disability over time may be the basic strategy to find out which factors contribute to a greater or lesser risk of disability and, thus, to promote strategies to a healthier aging process.

Body composition and cardiorespiratory fitness are the most studied health-related variables of physical fitness and function. Changes in body composition with a loss of muscle mass, bone mass, water content along with musculoskeletal and neuromotor function may predispose older adults to a functional decline with risk of disability increasingly high (5). In addition, changes in cardiorespiratory function with a decrement resulting as much as 50% by the age of 70 contribute also to a higher risk of disability (6). These previous findings have been partially confirmed in longitudinal studies focused on aging and disability, where independent associations of aging were found between lower mobility and mortality (7), and survival (8), dynapenia and abdominal obesity with disability worsening (9), cardiorespiratory fitness and muscle function (10), or cognitive/mental state or/and comorbidities and chronic diseases (11–13).

Although older adults are generally less active, physical activity and exercise training have beneficial effects on neuromuscular adaptations (muscle and myofiber hypertrophy with gains in strength and power (14)) in healthy older adults, depending on clinical status and the training modality exercise training, specially endurance training improves different aspects of muscle oxygenation (15). In fact endurance and resistance training programs have proved to be effective to the positive increment in aerobic capacity and thus also in functional capacity of participants as so in cognitive function and in the reduction of the risk for chronic diseases (16, 17). However, a great variability exists in functional responses to exercise and in levels of adherence and preferences (18). Particularly, changes in body composition with a loss of muscle mass, bone mass, and water content along with musculoskeletal and neuromotor function predisposed older adults to a functional decline with risk of disability increasingly high (5).

Despite this increment in research, it remains the need to identify the best predictors for functional capacity and what is the contribution of physical activity to maintain this capacity with aging.

It was our purpose to characterize longitudinally functional physical fitness in a sample of Portuguese community-dwelling older adults and find plausible associations between functional, and anthropometric variables, and differences across physical activity levels and cardiometabolic comorbidities in this population over a 3-year period.

## Materials and Methods

### Population

Participants were recruited for this longitudinal study in day care centers and senior universities and were eligible if they were older than 60 years and independent in activities of daily living (ADL). Participants were excluded if they were unable to ambulate without assistance. Body composition, functional fitness, and physical activity assessments were carried out in all participations who met the inclusion criteria at beginning of the study (M1) and 3 years later (M2).

After an explanation of the study and after a signed informed consent, in accordance with the Declaration of Helsinki, participants performed a battery of tests to assess functional physical fitness. All study procedures were approved by the institutional review boards of the participating institutions.

### Body Composition

Weight and height were measured using a digital balance with a stadiometer (SECA^®^). Body mass index (BMI) was calculated (BMI = kg/m^2^). An anthropometric tape was used for measuring waist (WC) and hip circumferences (HP) as markers of regional adiposity. Waist circumference was measured at the smallest circumference above the umbilicus and below the xiphoidal process (19) and hip circumference was measured at the highest circumference of the buttocks in a level horizontal plane. Ratio waist–hip was found by dividing the waist circumference (cm) by hip circumference (cm) (20, 21).

Body Composition was assessed using anthropometric measurements (height, weight, waist, and hip circumference) and BMI and waist to hip ratio (WHR) were calculated.

Whole-body skeletal muscle mass (SMM) was estimated using the following Al-Gindan et al. (22) equations:

(1) $$\begin{array}{c} \text{SMM}\left(\text{kg}\right)\text{in male}=39.5+(0.665\;\text{weight(kg)})-(0.185\;\text{WC}(\text{cm}))-(0.418\;\text{HC}\;(\text{cm}))\\ -(0.08\;\text{age}\;(\text{years})),r^{2}=0.79;\;\text{SEE}=2.7\;\text{kg}: \end{array}$$

and

(2) $$\begin{array}{c} \text{SMM}\left(\text{kg}\right)\text{in females}=2.89+(0.255\;\text{weight(kg)})-(0.175\;\text{HC}(\text{cm}))-(0.038\;\text{age}\;(\text{years}))\\ +(0.118\;\text{Height(cm)});r^{2}=0.59;\;\text{SEE}=2.1\;\text{kg} \end{array}$$

where WC, waist circumference, HC, Hip circumference.

### Functional Fitness and Disability

The Senior Fitness Test battery was used to assess functional capacity. This battery consists of six items to assess lower limbs strength and flexibility, upper body strength and flexibility, agility and dynamic balance, and aerobic endurance. All the tests were performed according to guidelines and protocols for administration (6). Briefly, lower limbs strength was measured with the 30 s chair stand test. An arm curl test was utilized for upper body strength assessment, which consists of carrying out as many forearm curls as possible within 30 s with 2.27 kg (5 lb) and 3.63 kg (8 lb) dumbbells for women and men, respectively. The chair sit-and-reach and the back scratch tests were used to assess lower and upper body flexibility, respectively. The 8-foot up and go tests were used to assess mobility. To measure aerobic endurance we used the 6-min walk test (6MWT) (23), and estimated distance walked was calculated with a validated equation (24).

(3) 6MWD(m)=218+(5.14Height)−(5.32Age)−(1.80Weight)+(51.31Sex),

where *F* = 0; *M* = 1; *r*^2^ = 0.66.

Isometric strength was assessed with a handgrip test using the American Society of Hand Therapists (ASHT) protocol. It was measured at both hands (in kg; JAMAR^®^ dynamometer). ASHT recommends a sitting position with shoulder adducted, elbow flexed at 90° for arm and wrist in neutral position and wrist between 0° to 30° of dorsal flexion (25).

Balance was assessed with the Berg Balance Scale (BBS) (26) because it is easy to use, it takes ~15–20 min to complete and is appropriate for clinical and community settings. It consists on 14 items that the subject must complete safely. Each item is scored from 0 (unable to complete the task) to 4 (independent and safe performance). Maximum score is 56 points (26).

In addition, participants completed and *ad hoc* questionnaire about comorbidities or non-communicable diseases and musculoskeletal pain conditions.

### Physical Activity

Levels of physical activity (LPA) were assessed and subjects were classified in sedentary/inactive, insufficiently active or active according the classification of physical behavior (19). An active participant was considered who perform 30 min of moderate activity at least 5 days a week and/or 20 min of vigorous activity 3 days per week or a combination of both.

### Statistical Analysis

For all statistical analysis, the IBM SPSS 21.0 package for Macintosh was used (SPSS Inc., IBM Company, Chicago, IL, USA). The confidence level was established with a statistically significant *p*-value of less than 0.05. All variables were inspected and confirmed for a non-normal distribution prior to analysis (Kolmogorov–Smirnov normality test). Basic characteristics of the participants were obtained by analyzing frequencies (%), or calculating mean and SD (mean ± SD).

Differences of performance, body composition, and functional fitness between M1 and M2 were analyzed by Wilcoxon signed-rank test. In addition, differences by sex were carried out using a Mann–Whitney *U* test.

The association between comorbidities and functional variables at M1 and M2 were analyzed with Wilcoxon Signed Rank test or a Mann–Whitney *U* test, for comorbidities with significant impact on functional variables.

Linear regression models were performed for the clinical variables that had significant differences between two measures (differences in clinical variables were dependent variables and physical fitness and body composition as independent). Finally, a Kruskal–Wallis analysis was conducted to find differences in functional variables between physical inactive, insufficiently active and active.

## Results

Baseline (M1) and 3 years later (M2) characteristics of participants aged between 60 and 91 years, all Caucasian, are described in Table 1.

**Table 1:** Variables of characterization of participants (12 males; 31 females; total *n* = 43) (mean ± SD) or *n* (%) for characterization of comorbidities.

	M1	M2	*p*
**Age (years)**			
M	73.50 ± 8.84	76.50 ± 8.85	**0.000**
F	73.94 ± 8.78	76.81 ± 8.68	**0.000**
T	73.81 ± 8.69	76.72 ± 8.62	**0.000**
**Weight (kg)^α^, ^β^**			
M	83.76 ± 12.93	81.67 ± 12.37	**0.031**
F	65.42 ± 7.69	64.54 ± 7.98	NS
T	70.54 ± 12.47	69.31 ± 12.08	**0.009**
**Height (m)^α^, ^β^**			
M	1.69 ± 0.08	1.69 ± 0.08	NS
F	1.53 ± 0.06	1.53 ± 0.06	NS
T	1.58 ± 0.09	1.57 ± 0.09	NS
**BMI**			
M	29.21 ± 4.05	28.59 ± 3.75	**0.028**
F	27.99 ± 3.36	27.69 ± 3.46	NS
T	28.34 ± 3.56	27.94 ± 3.52	**0.041**
**WHR^α, β^**			
M	0.96 ± 0.09	0.97 ± 0.10	NS
F	0.82 ± 0.06	0.85 ± 0.07	**0.04**
T	0.86 ± 0.09	0.88 ± 0.09	**0.003**
**SMM^α, β^**			
M	26.70 ± 6.51	24.51 ± 6.36	NS
F	16.31 ± 2.12	16.05 ± 1.95	**0.031**
T	19.21 ± 6.05	18.26 ± 5.18	**0.009**
**Diabetes type II [*n* (%)]**			
M	4 (33.3%)		
F	4 (12.9%)		
**Hypercholesterolemia [*n* (%)]**			
M	5 (41.7%)		
F	9 (29.0%)		
**Hypertension [***n***(%)]**			
M	11 (91.7%)		
F	21 (67.7%)		
**Cardiopulmonary conditions [*n*(%)]**			
M	8 (66.7%)		
F	11 (35.5%)		
**Musculoskeletal complaints [*n* (%)]**			
M	8 (66.7%)		
F	24 (77.4%)		
**Number of comorbidities [*n* (%)]**			
0	6 (14%)		
1	12 (27.9%)		
2	16 (37.2%)		
3	7 (16.3%)		
4	2 (4.7%)		

*M, male; F, female; T, total; BMI, body mass index (weightheight^2^); WHR, waist to hip ratio (circumference of the waist/circumference of the hip); SMM, skeletal muscle mass [according to equations in Al-Gindan et al. (22)]; M1, first moment of assessment; M2, second moment of assessment; p < 0.05 based on Wilcoxon signed-rank test for differences between M1 and M2, respectively, for males or females; α, β, p < 0.05 based on Mann–Whitney U test for differences between males and females on M1 and M2, respectively*.

*NS = Not Significant*.

Fourteen percent had a normal BMI at M1 but this percentage was increased to 21% at M2. There were not underweight cases and the prevalence of obesity was 23.3% (BMI > 30.0).

Only six participants reported none disease or musculoskeletal issue. Eighty six percent reported one to four comorbidities and 58.1% present multibordity (2 or more chronic conditions): type II diabetes was present in 18.6% of all the participants, hypercholesterolemia in 32.6%, and hypertension in 74.4%. Musculoskeletal complaints were present in 74.4% of participants. Overall, 33.3% participants suffered one or more falls in the previous year (at M1). Almost all the anthropometrical and body composition characteristics changed over the 3-year follow-up period (Table 1). Males reduced their weight and BMI while females decreased SMM and had small although significant increment in WHR (Table 1).

Most our participants were sedentary or inactive (37.2%) or insufficiently active (39.5%) and only 23.3% were active at M1. These latter results were similar at M2: 39.5% were sedentary, 30.2% insufficiently active, and 30.2% were active.

Regarding physical fitness capacity, there were only a few components with significant alteration over time (Table 2). Males reduced flexibility with a small increase in upper limb strength (arm curl). Among females, we could observe a decrease in balance and muscle strength. No changes in functional aerobic capacity variables were verified in both sexes (Table 2). Although 58.3% of males and 48.4% of females presented values for the handgrip that were classified as poor (19) at M1, only 8.3% of males and 12.9% of females presented values on handgrip strength that could be classified as having sarcopenia at M2.

**Table 2:** Values for functional fitness variables by gender (12 males; 31 females; total *n* = 43) (mean ± SD).

	M1	M2	*P* Wilcoxon
**6MWT (m)α**			
M	480.45 ± 98.38	479.67 ± 120.25	NS
F	394.21 ± 106.59	402.27 ± 117.13	NS
T	418.28 ± 110.37	424.38 ± 121.81	NS
**% of ED-6MWT**			
M	79.97 ± 11.99	81.51 ± 15.38	NS
F	78.91 ± 16.36	82.31 ± 19.01	NS
T	79.21 ± 15.14	82.08 ± 17.87	NS
**Chair sit-and-reach test (cm)**			
M	−4.13 ± 9.11	−9.58 ± 11.12	NS
F	−1.31 ± 9.42	−5.03 ± 9.68	NS
T	−2.09 ± 9.31	−6.30 ± 10.18	**0.015**
**Back scratch test (cm)**			
M	−14.00 ± 14.37	−18.83 ± 16.41	**0.041**
F	−10.15 ± 10.03	−13.47 ± 12.89	NS
T	−11.22 ± 11.36	−14.96 ± 13.96	**0.006**
**30 s chair stand test**			
M	13.33 ± 2.71	14.08 ± 4.29	NS
F	11.35 ± 3.68	12.80 ± 3.80	**0.008**
T	11.91 ± 3.52	13.19 ± 3.94	**0.008**
**Arm curl test**			
M	14.58 ± 2.78	17.17 ± 5.41	**0.029**
F	13.26 ± 4.19	14.71 ± 4.00	**0.015**
T	13.63 ± 3.86	15.40 ± 4.51	**0.001**
**Right handgrip (kg)^α, β^**			
M	38.58 ± 9.34	38.58 ± 9.98	NS
F	26.13 ± 7.09	25.74 ± 6.31	NS
T	29.60 ± 9.53	29.33 ± 9.41	NS
**Left handgrip (kg)^α, β^**			
M	37.08 ± 11.52	36.29 ± 10.78	NS
F	23.06 ± 5.73	23.35 ± 5.44	NS
T	26.98 ± 9.94	26.97 ± 9.28	NS
**8-foot up-and-go test (s)**			
M	6.66 ± 1.54	6.85 ± 2.25	NS
F	8.27 ± 2.89	8.47 ± 3.76	NS
T	7.83 ± 2.67	8.01 ± 3.45	NS
**Berg scale**			
M	54.33 ± 1.72	51.83 ± 4.47	NS
F	52.74 ± 3.54	49.87 ± 6.18	**0.000**
T	53.19 ± 3.20	50.42 ± 5.77	**0.000**

*Legend: M, male; F, female; T, total; 6MWT, 6 min walk test; % of ED-6MWT, percentage of the estimated distance on 6MWT; p < 0.05 based on Wilcoxon signed-rank test for differences between M1 and M2, respectively, for males or females; α, β, p < 0.05 based on Mann–Whitney U test for differences between males and females on M1 and M2, respectively*.

*NS = Not Significant*.

Balance assessed by BBS was significantly reduced only in women. Nevertheless, a change of 6 points in BBS score indicated with 95% of confidence that a genuine change in function has occurred (27) and this occurred for 18.6% of our sample (three males and five females).

Women reduced lower limbs (30 s chair stand test) strength at M2 but not mobility, although they took longer to complete the test, the decrement in mobility was not significant (8-foot-up-and-go test). BBS results informed that our participants have an increased risk of 43% of falling, 16% risk for suffering multiple falls, and 24% risk for suffering an injurious fall.

Table 3 shows the differences on functional variables between diabetic and non-diabetic groups, and participants with or without hypercholesterolemia at M1 and M2 (only for those variables with significant differences at least in one time point). In summary, only type II diabetes and cholesterolemia conditions seemed to influence the performance in endurance capacity, Berg scale and flexibility (type diabetes), and strength (hypercholesterolemia). Longitudinal changes in endurance capacity (6MWT) were reduced in diabetic participants but not in non-diabetic ones (Table 3).

**Table 3:** Values for functional fitness variables related with comorbidities (12 males; 31 females; Total *n* = 43) (mean ± SD).

	M1	M2	*P* Wilcoxon
**Diabetes Mellitus**			
6MWT (m)			
Non-diabetes (*n* = 35)	431.35 ± 113.04	442.118 ± 123.85	NS
Diabetes (*n* = 8)	361.09 ± 80.25	349.01 ± 81.07	NS
*U* Mann–Whitney	NS	**0.026**	
**Back Scratch Test (cm)**			
Non-Diabetes (*n* = 35)	−8.84 ± 10.26	−12.38 ± 13.05	**0.027**
Diabetes (*n* = 8)	−21.63 ± 10.51	−26.25 ± 12.82	NS
*U* Mann–Whitney	**0.006**	**0.005**	
**Berg scale**			
Non-Diabetes (*n* = 35)	53.54 ± 3.14	51.11 ± 5.91	**0.000**
Diabetes (*n* = 8)	51.63 ± 3.20	47.38 ± 4.14	NS
*U* Mann–Whitney	**0.033**	**0.022**	
**Hypercholesterolemia**			
Left handgrip (kg)			
Non-High Cholesterol (*n* = 29)	24.38 ± 6.09	24.22 ± 6.34	NS
High Cholesterol (*n* = 14)	32.36 ± 13.89	32.64 ± 11.28	NS
*U* Mann–Whitney	NS	**0.045**	

*6MWT, 6 min’ walk test; p < 0.05 based on Wilcoxon signed-rank test for differences between M1 and M2, respectively, for comorbidities; p < 0.05 based on Mann–Whitney test for differences between M1 and M2, respectively, for comorbidities*.

*NS = Not Significant*.

Anthropometrical and functional variables were statistically different across LPA and a better profile in functional capacity and anthropometry was showed for active when compared with inactive participants (Table 4). Distribution of comorbidities was similar among the physical activity level groups.

**Table 4:** Kruskal–Wallis analysis for differences in anthropometrical and functional variables between physical inactive, insufficiently active and active participants.

	Inactive (*n* = 17), mean ± SD	Insufficiently active (*n* = 13), mean ± SD	Active (*n* = 12), mean ± SD	*p*-Value
BMI (kg/m^2^)	28.64 ± 3.53	27.92 ± 4.34	27.26 ± 2.79	NS
WHR	0.89 ± 0.11	0.88 ± 0.07	0.87 ± 0.10	NS
SMM (kg)	16.42 ± 4.00	19.47 ± 7.04	19.56 ± 4.08	**0.032**
6MWT (m)	334.12 ± 109.64	449.07 ± 86.63	525.50 ± 71.83	**0.000**
% of ED-6MWT (%)	72.32 ± 20.02	84.27 ± 13.53	93.54 ± 10.54	**0.006**
Chair sit-and-reach test (cm)	−9.65 ± 10.99	−5.65 ± 9.56	−2.57 ± 8.86	NS
Back scratch test (cm)	−22.23 ± 13.77	−8.76 ± 11.23	−11.65 ± 13.23	**0.011**
30 s chair stand test (repetitions)	10.88 ± 3.40	14.46 ± 3.47	14.92 ± 3.77	**0.009**
Arm curl test (repetitions)	12.65 ± 2.91	17.54 ± 5.12	16.85 ± 3.95	**0.004**
Right handgrip (kg)	23.29 ± 6.29	32.85 ± 9.90	33.69 ± 8.55	**0.003**
Left handgrip (kg)	21.12 ± 5.90	31.62 ± 10.92	29.96 ± 7.33	**0.001**
8-foot up-and-go test (s)	10.49 ± 3.96	6.59 ± 1.81	6.01 ± 1.27	**0.000**
Berg scale	46.29 ± 6.54	52.77 ± 3.46	53.46 ± 2.69	**0.001**

*BMI, body mass index; WHR, waist to hip ratio; SMM, skeletal muscle mass; % of ED-6MWT, percentage of the estimated distance on 6MWT. p < 0.05; NS = Not Significant*.

Finally, the linear regression models showed the best predictors of functional tests.

The first linear regression model to the dependent variable “Berg Balance Scale” was associated with 6MWT and “Right Handgrip” adjusted to weight was significant (*F* = 27.076; *p* = 0.000; *R*^2^ = 0.681, and SEE = 3.400). The contribution of each predictor to the model was significant with a standardized Beta coefficient of 0.635 (*p* = 0.000) and 0.313 (*p* = 0.039), respectively. The whole model explained 68% of the variance.

The second linear regression model to the dependent variable “30 s chair stand test” was associated with 6MWT adjusted to weight (*F* = 26.192; *p* = 0.000; *R*^2^ = 0.567, and SEE = 2.658). The contribution of the predictor to the model had standardized Beta coefficient of 0.779 (*p* = 0.000) with 57% of the variance explained by the model.

“Arm curl test” was correlated with 6MWT and “Left Handgrip” adjusted to weight (F = 12.190; *p* = 0.000; R2 = 0.484 and SEE = 3.362). The contribution of each predictor to the model was significant with a standardized Beta coefficient of 0.406 (*p* = 0.009) and 0.416 (*p* = 0.024), respectively (the model explained 48% of the variance).

Lower flexibility (“Chair Sit-and-Reach Test”) was associated with upper flexibility (“Back Scratch Test”) (*F* = 9.111; *p* = 0.001; *R*^2^ = 0.313, and SEE = 8.646). The contribution of the predictor to the model was significant with a standardized Beta coefficient of 0.529 (*p* = 0.000) and the model explained 31% of the variance. Regarding, upper flexibility (“Back Scratch Test”) was correlated with BBS (*F* = 6.894; *p* = 0.003; *R*^2^ = 0.256, and SEE = 12.34) The contribution of the predictor to the model was significant with a standardized Beta coefficient of 0.500 (*p* = 0.001) and the model explained 26% of the variance.

Summarizing, physical function predictors, 6MWT and handgrip strength were the best predictors.

### Alternative or Additional

Finally, we could not find associations between changes in functional capacity tests and Berg scale or improvement in comorbidities.

## Discussion

To better understand the complex nature of physical function decline among older adults, we pursued two directions. First, we evaluated and identified the progression of physical function decline over a 3-year follow-up in a sample of people older than 60 years. Our results showed differences between gender in the anthropometric and functional variables after follow-up period, and these differences could be associated with changes in body composition across the aging process which is not a new finding. The level of functional fitness decreased with age, and the decrease was more important in females. On the other hand, this prospective longitudinal study showed the association between functional variables related to the balance, risk of falls, strength, physical fitness tests, and comorbidities. Our data bring knowledge to understand the variables that suffer the worst decrease with aging and may contribute to create more efficient strategies focused in these functional variables. In this scenario, the most important finding in this study is the relevance of 6MWT as predictor of other functional capacities and the influence of type II diabetes in 6MTW performance and age-related impairment.

The specific sexual phenotype of change for body weight was not new (BMI was reduced for male participants, but not for females); this adaptation followed the sarcopenic obesity paradigm since women did not lose weight in 3 years, but they increased waist and hip circumference (regional adiposity markers), and decreased whole-body skeletal muscle mass (SMM), which may increase the risk of hip fractures in the presence of a loss of balance. Previous studies (28, 29) concluded that greater abdominal obesity measured by WHR increased the risk of hip fracture considerably. The mechanism for an increased fracture risk with increasing WHR after adjusting for BMI might be mechanical instability and impaired balance induced by an increased body size in the abdominal region, leading to a fall (30). It has been suggested that carrying excess weight on the torso rather than on the hips increases the risk of falls that result in hip fracture, perhaps due to a higher center of gravity (28). This paradigm that explains a relationship between the increasing in the WHR and increment in the risk of falls was partially confirmed with our results of differences in the BBS variables, which suffered biggest significant changes (see Table 2). Other previous studies have investigated the relationship between obese and the risk of falls and fear of falls in older women (31–33). In general, results from our study are aligned with previous studies (34–37) and also with the study of Bird et al. (38) were balance decreased without loss of strength.

The influence of physical activity in functional capacity was confirmed in our group of age with a dose–response effect, so LPA were positively associated with performance in functional tests showing better results for those who are more active. Although only 30% of the participants accomplish physical activity guidelines (physical activity at least three times a week) the deleterious effect of PA on physical function impairment may not be related with a fixed quantity of PA, which may indicate that even low amounts of PA must promote significant benefits for physical function.

Our data highlighted that balance seems to be greatly explained by aerobic functional capacity (6MWT) and strength (handgrip). These results are in accordance with a previous study, which has shown that higher lower-limb function, balance, and mobility are associated with better walking ability and distance covered in the 6MWT by healthy older adults (39). Our study revealed that 6MWT and ha handgrip strength are also related with the BBS. Handgrip strength has been used as an outcome to measure muscle strength because it has been identified as strongly related with lower extremity muscle power, knee extension torque, and calf cross-sectional muscle area (40, 41) and could predict functional decline (42, 43). These findings reveal not only major relevance of cardiorespiratory fitness but also strength for preserving a good functional capacity. The main result from functional capacity were those related with comorbidities, although functional capacity were barely affected by group of comorbidities, so type II diabetes seemed to affect functional capacity (lower distance on 6MWT, lower flexibility, and lower balance than participants without diabetes type II). Furthermore participants with diabetes present a higher decrement on distance walked on 6MWT, which can potentiate the functional decline and risk of disability associated with aging. This topic should be object of more studies since this association may be a determinant of a frailty phenotype (44–46). In addition, we noticed that 39.5% at M1 and 35.7% of participants at M2 walked less than 400 m on 6MWT, which is a cut-off point to classify sarcopenia with limited mobility; on average, diabetic participants walked way below of this cut-off latter, representing their high susceptibility to be classified as sarcopenic obese and higher risk of frailty (47, 48). Regarding handgrip data, values split by hypercholesterolemia group were paradoxical since hypercholesterolemia group had higher values of strength, nevertheless the proportion of males and body weight values in the group of hypercholesterolemia were higher than in the group without hypercholesterolemia (35.7 vs 24.9%)., which may explain our results. Although the fact of having or not having multimorbidity [the presence of 2 or more chronic conditions (49)] may put older people in higher risk of frailty our results did not confirm this hypothesis.

Physical decline, including muscle weakness, balance dysfunction and mobility problems are related to functional decline, contributing to limit functional capacity while performing ADL and increasing the risk of falling (50, 51). Individualized intervention programs should address fall risks previously identified and they should include balance training, aerobic endurance, muscle strengthening, and flexibility (50).

Improvements in balance may rely in specific balance training programs (38) but also in other strategies. Our results may suggest that exercise prescription for older people should include also aerobic endurance and muscle training (50) as guidelines state, with recommendations to walk at least 30 min a day (52). Physical activity such as walking in different environments should be prescribed as an activity of daily living with benefits on physical capacity especially in balance and specially to those that do not meet the recommended 150 min per week of physical activity of moderate intensity and have or are at risk for chronic diseases (53), with special attention to groups of participants with comorbidities such as diabetes.

### Strengths and Limitations

This was a longitudinal study that analyzed whether aging-related changes could be related with a lower decrement in functional variables such as muscle strength, flexibility, or mobility. However, the time interval was too short to found undeniable or high predictive value in variables of disability.

Although our study has a well-defined target population (day care centers and senior universities), participants were volunteers, and this could be a small bias which could be overcome by a larger sample. In addition, the small size of the sample is also a limitation that prevents a more accurate analysis of the results.

A higher time interval and a larger sample should be considered in future studies.

## Conclusion

Results of this 3-year follow-up study confirmed the relevance of endurance and strength capacities, which may be improved across physical activity levels in combination with other body composition and physical function variables. The main relevance was the influence of type II diabetes 6MWT impairment in the second time point, which may confirm the deleterious consequences of the disease on cardiovascular capacity and sarcopenia; in addition, these results must provide construct validity of 6MWT. Finally, our results suggested that the loss of functional capacity must be more related with qualitative variables than morphological (for example, SMM) since we could not find any relationship between changes in body composition and physical function variables.

When prescribing exercise or programs for promoting health targeting this population greater attention should be given in walking activities and activities that use hands since these specific components seems to have a major impact in functional physical condition.

## Ethics Statement

After an explanation of the study, participants signed an informed consent. All procedures were in accordance with the Declaration of Helsinki. All study procedures were approved by the institutional review boards of the participating institutions.

## Author Contributions

MTT, AG-M, EC, and BF have equally contributed to this manuscript, in designing the study. Data were collected by MTT, AG-M, and BF and were analyzed by all authors. Data interpretation and manuscript preparation were undertaken by MTT, AG-M, EC, and BF. All authors approved the final version of the paper.

## Conflict of Interest Statement

The authors declare that the research was conducted in the absence of any commercial or financial relationships that could be construed as a potential conflict of interest.
